# Influence of Oral and Maxillofacial Radiology Training and Experience on Attitudes Toward Artificial Intelligence in Dentistry: A Cross-Sectional Questionnaire Study

**DOI:** 10.3390/healthcare14131907

**Published:** 2026-07-01

**Authors:** Ersen Bilgili, Ezgi Tokgöz, Nilay Dalgan Ayaz, Göksu Derinsu, Gülnihal Güneş

**Affiliations:** Department of Oral and Maxillofacial Radiology, Faculty of Dentistry, Izmir Katip Celebi University, Izmir 35640, Turkey; ezgi.tokgoz@ikc.edu.tr (E.T.); nilay.dalganayaz@ikc.edu.tr (N.D.A.); goksu.ozdemir@ikc.edu.tr (G.D.); gulnihal.gunes@ikc.edu.tr (G.G.)

**Keywords:** artificial intelligence, dentistry, oral and maxillofacial radiology, survey, professional experience, dental education

## Abstract

**Objective**: To evaluate whether professional status and training level influence attitudes toward artificial intelligence (AI) in dentistry, and to compare present-day AI attitudes with expectations for near-future AI applications. **Materials and Methods**: This cross-sectional questionnaire study drew on responses from 234 volunteers spanning four professional groups: 63 fourth-year dental students, 78 fifth-year dental students, 47 oral and maxillofacial radiology residents, and 46 oral and maxillofacial radiology specialists/faculty members. Present-attitude items, near-future-attitude items and awareness of selected AI applications were evaluated using multivariable models adjusted for professional status, sex, and age. **Results**: Internal consistency was high for both the present-attitude subscale (alpha = 0.852) and the near-future-attitude subscale (alpha = 0.872). Near-future ratings exceeded present-day ratings in all five paired domains (all *p* < 0.001). Professional status/training level was significantly associated with the present-attitude composite score (*p* = 0.017) and with the change score between near-future and present attitudes (*p* < 0.001), but not with the near-future composite score in isolation (*p* = 0.208). Radiology residents showed lower present-attitude scores (β = −2.41) yet a larger change score (β = +1.95) relative to fourth-year students. **Conclusions**: More experienced radiology groups were found to be relatively more cautious but more aware of certain present-day applications, yet showed a stronger tendency toward accepting near-future AI capabilities—particularly for structured, workflow-oriented tasks. Sustainable AI integration in dentistry may require more than attitudinal readiness—it may depend on grounded familiarity with validated tools and on addressing the practical and economic realities of clinical implementation.

## 1. Introduction

Artificial intelligence (AI) is defined as the intelligence exhibited by computers and computer-controlled robots. When John McCarthy first proposed the term “artificial intelligence” in 1955, he described it as the science and engineering of making intelligent machines—systems capable of performing tasks that would ordinarily require human reasoning and judgment [1]. Nearly seven decades later, that founding vision has moved from theoretical ambition to clinical reality, with implications that now reach directly into how diagnostic information is generated, interpreted, and acted upon across medicine and dentistry.

Mesko’s oft-cited characterization of AI as “the stethoscope of the 21st century” [2] has grown steadily less metaphorical over the past decade. In dentistry, AI applications have moved well beyond proof-of-concept demonstrations and now feature in research on diagnostics, treatment planning, workflow optimization, and data management [3,4,5,6,7,8,9].

In medical imaging, the push toward AI has been driven above all by a straightforward clinical need: achieving greater diagnostic throughput with improved accuracy, using the same or fewer resources. When AI components are embedded in an imaging workflow, they can reduce error rates and deliver pre-analyzed images with flagged findings, easing the cognitive burden on clinicians while keeping human oversight in the loop. Accordingly, both research programs and health policy initiatives have turned increasing attention to supporting AI development in this area [10].

Radiology lends itself particularly well to these developments. Most image-based tasks ultimately come down to quantifying radiographic features—measuring, detecting, characterizing, and tracking patterns across time and populations—and these are precisely the kinds of tasks machine learning handles well. From the early computerization of radiology in the 1980s onward, the field has progressively shifted from subjective visual interpretation toward more objective, reproducible analysis, and the pace of that shift has accelerated in step with data volumes and computational capacity [10].

Within dentistry, oral and maxillofacial radiology (OMFR) has emerged as the specialty most visibly engaged with AI, and for understandable reasons: classification, detection, segmentation, and structured decision-support are tasks well-suited to machine-learning approaches [5,6,7,8,11,12]. Published reviews confirm that AI models are already in development for image interpretation, image enhancement, and automated analysis in dentomaxillofacial radiology—though clinical uptake remains uneven and the evidentiary base is still maturing [5,6,13,14].

Technical capability alone, however, will not determine how widely AI is adopted in clinical practice. Uptake also depends on whether practitioners trust it, understand it, and feel prepared to use it responsibly [15,16,17,18,19,20,21,22]. On this front, the educational literature has become increasingly precise: dental curricula are increasingly called upon to move beyond introductory AI definitions and to cover model evaluation, generalizability, explainability, accountability, and governance [17,18,19,20].

While technical performance remains a primary driver of AI integration, the implementation landscape is increasingly governed by regulatory frameworks designed to ensure safety, transparency, and accountability. Recent initiatives, such as the EU AI Act, establish a classification system for AI tools based on risk, directly impacting how diagnostic software must be validated and documented before clinical deployment. Understanding these regulatory prerequisites is essential for practitioners, as it bridges the gap between proof-of-concept AI performance and real-world clinical reliability [17].

The rapid evolution of artificial intelligence in dentistry necessitates a robust understanding of both technological capabilities and their clinical limitations. As highlighted by Zafar et al., the integration of AI tools is transforming traditional diagnostic workflows, yet their successful implementation relies on evidence-based practices that bridge the gap between algorithmic potential and chairside application [23]. Furthermore, as discussed by Hellesø and Røssberg, the challenge for modern healthcare providers is to integrate these machine learning-driven insights into a standard of evidence-based practice, ensuring that AI-generated clinical suggestions are both transparent and validated through rigorous oversight [24].

This is especially pertinent for OMFR, where the clinician’s role is not simply to accept an automated output but to critically appraise it within a broader diagnostic and medicolegal framework [5,6,13,19,20].

The existing literature on AI attitudes in dentistry and oral radiology is not small, but it is uneven. Surveys from India, Brazil, and Türkiye focusing on oral radiology have documented broadly optimistic views about AI’s future diagnostic role, and larger dental surveys and professional seniority. South Korea and Türkiye have shown that perceptions shift with educational background and seniority. What is harder to find is a study that follows this gradient continuously—from undergraduate dental students through OMFR residents to OMFR specialists and faculty—within a single analytically rigorous framework. There is also a notable gap in how prior studies handle the distinction between what practitioners think AI can do right now and what they expect it to be capable of in the near future. That distinction matters especially in OMFR, where diagnostic responsibility and workflow integration are already real rather than hypothetical. Without it, AI attitude scores risk conflating present-day skepticism with genuine pessimism about AI’s longer-term trajectory [9,25,26,27,28,29,30].

The present study was designed to address these gaps. Our primary aim was to evaluate whether professional status and training level in OMFR—alongside sex and age—influence AI-related attitudes, and to determine whether present-day attitudes and near-future expectations follow different patterns across the training spectrum. We hypothesized, as a null, that radiology education and experience would not be associated with AI attitudes.

## 2. Materials and Methods

### 2.1. Study Design

This cross-sectional questionnaire study recruited volunteers with varying levels of dental education and clinical experience. Participants were grouped into four categories: fourth-year dental students, fifth-year dental students, oral and maxillofacial radiology residents, and OMFR specialists and faculty members.

The rationale for forming these groups was grounded in the progressive stages of radiology education and clinical exposure. Fourth-year students had largely completed their theoretical training in oral diagnosis and radiology but possessed limited clinical experience; fifth-year students combined theoretical knowledge with initial clinical practice; OMFR residents had advanced radiology training and more substantial clinical exposure; and specialists and faculty members represented the highest level of expertise. General dentists and practitioners from other departments were excluded, as the study was designed to derive greater relevance and interpretive value specifically among those engaged in both the theoretical and practical dimensions of radiology.

Although the survey questions were developed by reviewing similar studies, they were largely shaped by the authors’ ideas. Examining the current perspective on artificial intelligence (AI) and how it might evolve in the near future allowed participants to engage in detailed and thoughtful reflection. Sections such as awareness of selected AI applications, future expectations, and the budget that could be allocated to an ideal professional AI application aimed to provide a social perspective alongside clinical effects. The survey was pilot-tested with five participants from each group and was published after being reviewed for errors and comprehension difficulties. Furthermore, this cross-sectional study should be evaluated not as a “psychometric scale with full validation,” but rather as a “domain-oriented survey.”

Participation was voluntary, and all participants provided electronic informed consent before the survey opened. The questionnaire was constructed using Google Forms and remained accessible for a two-week period. The survey link was distributed through the email lists of the Association of Oral and Maxillofacial Radiology, as well as through WhatsApp and other social media platforms. All participants were affiliated with dental faculties of Turkish universities.

**Figure 1:**
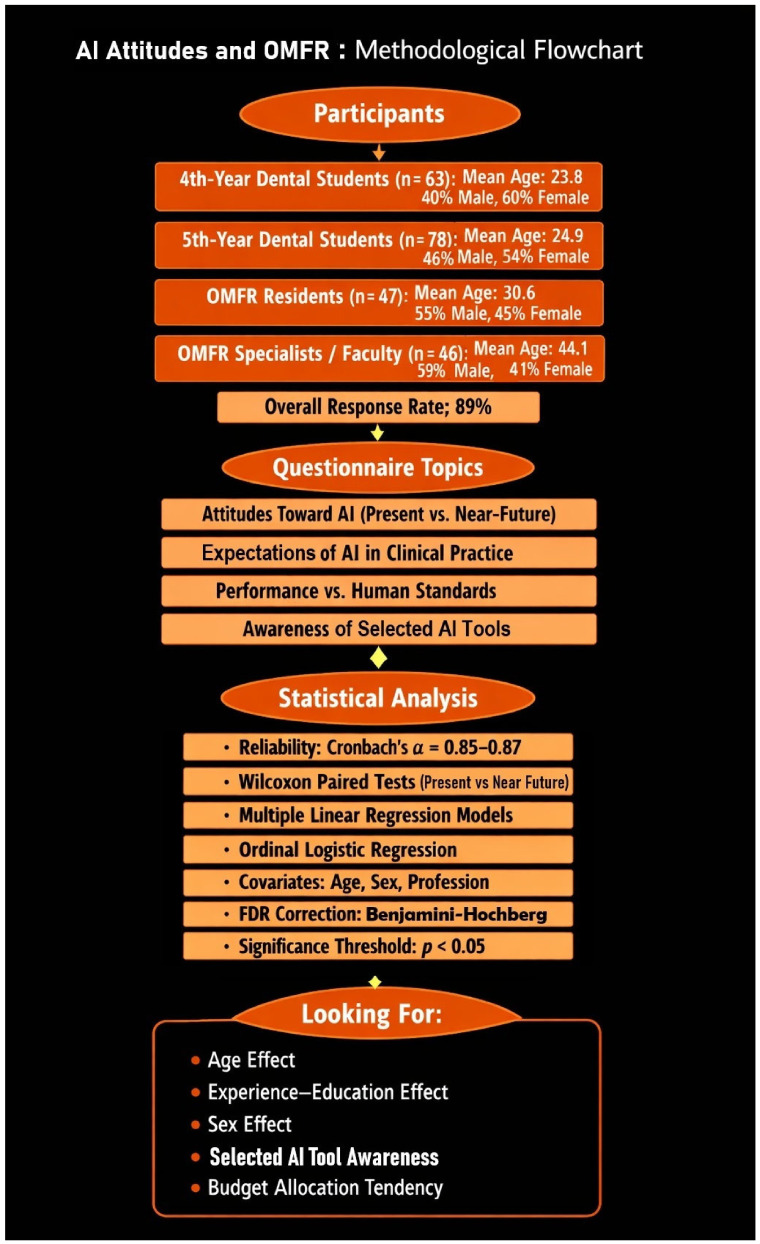
Flow diagram of the study.

Professional status/training level was the primary independent variable, operationalized across the four groups described above. Sex and age were included as additional predictors. The survey comprised 18 items organized into four sections: demographic information, AI awareness, views on the potential role of AI in oral diagnosis and radiology practice, and broader perceptions of AI’s present and near-future contribution to dentistry (Figure 1, Table 1).

Because professional status was the primary predictor and sex and age were planned covariates, the required sample size was estimated using an a priori power analysis based on a fixed-model, multiple linear regression framework. The effect size (Cohen’s f^2^ = 0.0625) used to calculate the sample size could not be derived from a directly comparable OMFR-specific attitude-toward-intelligence (AI) study. It was selected as a conservative, small-to-moderate effect size assumption (f^2^ = 0.02 for small, f^2^ = 0.15 for medium, and f^2^ = 0.35 for large). With an α of 0.05, 80% power, three degrees of freedom for the professional status block, and two additional predictors, the minimum required sample size was 179 participants. The final sample size of 234 exceeded this estimate, yielding an achieved power estimate of 0.902.

Since no formal content-validity index or full psychometric validation of survey was performed, the present study was designed as a domain-oriented survey rather than a formal psychometric scale; the results should therefore be read with caution.

### 2.2. Statistical Analysis

For the attitude analyses, item responses were coded on ordinal scales from 0 to 4, with higher values indicating more favorable attitudes toward AI, greater perceived feasibility, higher expected performance, greater awareness, or greater willingness to invest, depending on the item. The present-attitude block comprised Q1–Q5 and the near-future-attitude block comprised Q6–Q10. The perceived-feasibility block comprised Q11–Q15, the expected-success block comprised Q16a–Q16e, awareness of existing dental AI tools comprised Q17a–Q17e, and willingness to allocate a budget to AI comprised Q18.

Paired comparisons between present-day items and their near-future counterparts (Q1 vs. Q6, Q2 vs. Q7, Q3 vs. Q8, Q4 vs. Q9, and Q5 vs. Q10) were performed using the Wilcoxon signed-rank test. In these analyses, zero-difference pairs were excluded, and the reported W statistic corresponds to the smaller of the positive and negative signed-rank sums.

Composite-score outcomes—present-attitude composite, near-future composite, and change score—were analyzed using multiple linear regression models with professional status as a categorical predictor and sex and age as covariates. Global effects were evaluated using Type III F tests. Item-level ordinal outcomes were analyzed using ordinal logistic regression with the same predictors. Inferential families were defined a priori: the five paired present-versus-near-future comparisons formed one family, the composite-score models a second, and the item-level ordinal models a third. False discovery rate (FDR) correction following the Benjamini–Hochberg procedure was applied within each family where appropriate. Two-sided *p* values below 0.05 were considered statistically significant.

## 3. Results

The final sample comprised 234 participants, including 140 females (59.8%) and 94 males (40.2%). The dataset included 63 fourth-year students (26.9%), 78 fifth-year students (33.3%), 47 radiology residents (20.1%), and 46 radiology specialists/faculty members (19.7%). Response rates were highest among students (93.3%) and lowest among radiology residents (72.3%; Table 2). All participants completed the survey without missing data.

Internal consistency was high for both attitude blocks. Cronbach’s alpha reached 0.852 for the present-attitude block (Q1–Q5) and 0.872 for the near-future block (Q6–Q10). Corrected item-total correlations ranged from 0.588 to 0.740 for Q1–Q5 and from 0.585 to 0.748 for Q6–Q10, and removing any single item did not improve overall alpha (Table 3).

To evaluate the perception of artificial intelligence (AI) applications among different professional and educational levels, participants were stratified into four groups: 4th-year undergraduate students (Group 1), 5th-year undergraduate students (Group 2), radiology residents (Group 3), and radiology specialists/faculty members (Group 4). Descriptive statistics indicated a progressive trend toward more favorable perceptions of AI integration as clinical experience and educational level increased. The overall mean scores were 1.56 ± 0.22 for Group 1, 1.86 ± 0.28 for Group 2, 2.39 ± 0.31 for Group 3, and 2.76 ± 0.35 for Group 4 (Table 4). Given the heterogeneity of variance between the groups and the unequal sample sizes, the Welch ANOVA test was employed to assess differences in mean scores across the four groups. This approach was chosen to ensure robustness against violations of the homogeneity of variance assumption. Subsequent to the significant ANOVA results (F(3, 42.1) = 9.84, *p* < 0.001), Games–Howell post hoc tests were performed to conduct pairwise comparisons. This method was specifically selected due to its reliability in maintaining control over Type I error rates in the presence of unequal variances and unbalanced group sizes (Table 5).

**Figure 2:**
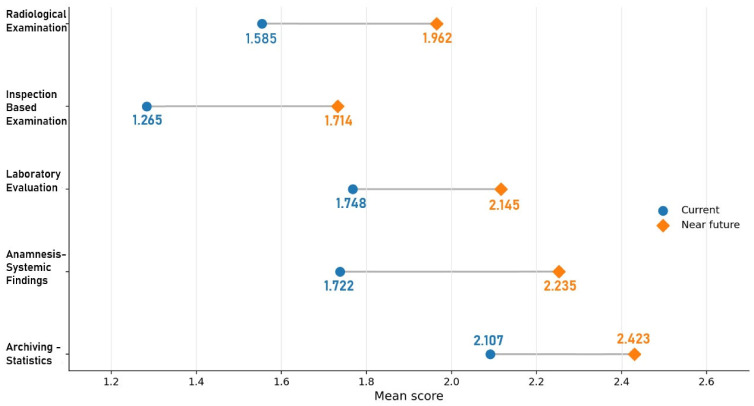
Dumbbell plot comparing mean current and near-future attitude scores across five domains. Points represent means.

Without exception, near-future ratings exceeded present-day ratings across all five domains. For radiologic examination, the mean score rose from 1.585 to 1.962; for inspection-based examination, from 1.265 to 1.714; for laboratory evaluation, from 1.748 to 2.145; for anamnesis/systemic findings, from 1.722 to 2.235; and for archiving/statistics, from 2.107 to 2.423 (Figure 2). Wilcoxon signed-rank tests confirmed statistically significant differences for all five domains (all *p* < 0.001). Because the questionnaire used a 5-point ordinal structure, many paired comparisons included zero differences; these were excluded from the Wilcoxon procedure, and the reported W values represent the smaller signed-rank sum. The number of non-zero paired differences ranged from 82 to 111 across domains, indicating that a substantial proportion of participants gave identical present and near-future ratings (Table 6).

**Figure 3:**
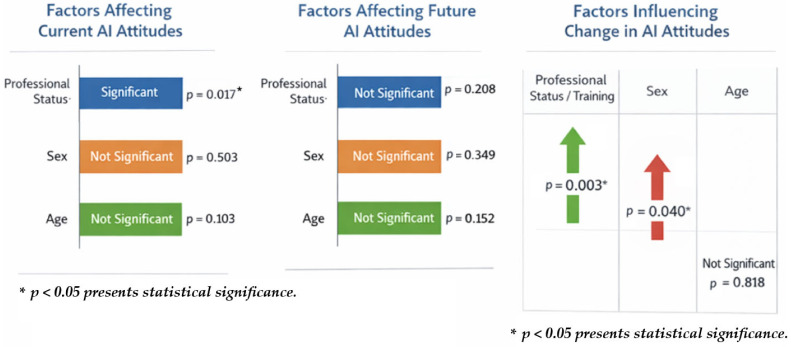
Factors influencing attitude towards AI. Regression results of professional status in OMFR, sex and age affecting present AI attitudes, near-future attitudes and the change between.

After adjusting for sex and age, professional status was significantly associated with the present-attitude composite score (F(3, 228) = 3.45, *p* = 0.017) and with the change score between near-future and present attitudes (F(3, 228) = 7.37, *p* < 0.001), but not with the near-future composite score alone (F(3, 228) = 1.53, *p* = 0.208). Compared with fourth-year students, radiology residents had a lower present-attitude composite score (β = −2.41, 95% CI: −3.96 to −0.86, *p* = 0.003), and radiology specialists and professors also scored lower on this composite (β = −2.65, 95% CI: −5.10 to −0.21, *p* = 0.017). The change score, however, was higher among radiology residents (β = 1.95, 95% CI: 0.73 to 3.16, *p* = 0.002). Sex was associated with the change score (β = 0.79, 95% CI: 0.04 to 1.54, *p* = 0.040), whereas age was not significantly associated with any composite outcome (Table 7, Figure 3).

After FDR correction, a limited number of item-level associations remained significant. For each item-level ordinal logistic regression model, the proportional odds assumption was assessed using a parallel-lines/Brant-type test. Model fit was evaluated by likelihood-ratio χ^2^ tests comparing the final model with the intercept-only model. Adjusted odds ratios with 95% confidence intervals were reported for predictors retained after FDR correction (Table 8). Professional status remained associated with Q2, Q3, Q7, Q11, Q14, Q15, and Craniocatch awareness; sex remained associated only with Q18. No age effect survived correction.

**Figure 4:**
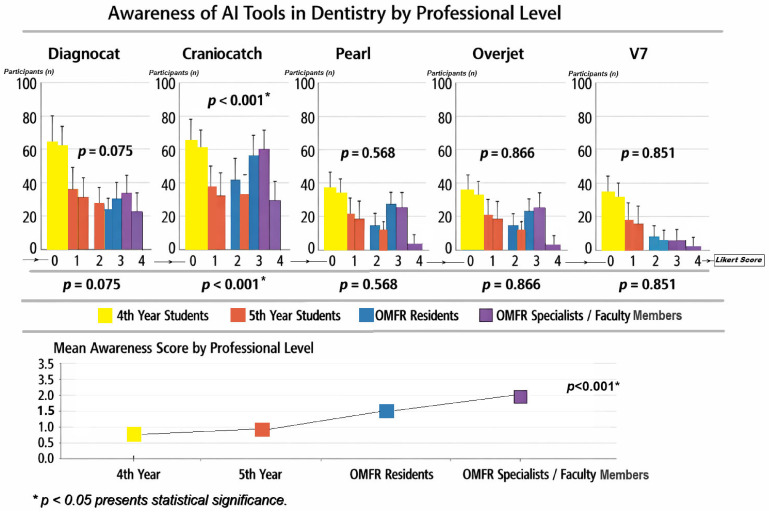
Awareness scores for selected dental AI applications according to professional status/training level.

Awareness of selected dental AI applications varied clearly across platforms. Considering the inequal number of participants, Craniocatch showed the highest overall recognition (mean 1.35 ± 1.52), followed by Diagnocat (1.15 ± 1.23), while Overjet (0.47 ± 0.90), Pearl (0.35 ± 0.80), and V7 (0.30 ± 0.75) remained low. Practical familiarity (score ≥ 3) was highest for Craniocatch (35.5%) and Diagnocat (21.4%), with the remaining applications below 6%. Reported actual use was confined mainly to Craniocatch (8.5%) (Table 9 and Table 10; Figure 4).

For Q18, the most common response was $11–50/month (34.6%), followed by $100+/month (23.5%) and $51–100/month (22.2%). Only 5.1% of respondents would allocate no budget. In adjusted analysis, willingness to allocate a higher budget was associated with sex (*p* = 0.001, FDR q = 0.028) but not with professional status or age. The $100+/month category was selected by 40.4% of males and 12.1% of females (Table 11).

## 4. Discussion

### 4.1. Methodological Considerations

The present cross-sectional questionnaire design enabled systematic comparison across four professional strata, ranging from undergraduate dental students to oral and maxillofacial radiology (OMFR) specialists or faculty members. The achieved sample size (*n* = 234) exceeded the a priori requirement, yielding adequate statistical power (0.902) and thereby enhancing the reliability of the findings. Internal consistency was high for both the present-attitude (α = 0.852) and near-future attitude (α = 0.872) scales, confirming the robustness of the measurement instrument.

Beyond the influence of demographic factors such as age and gender, the study sought to maintain the relevance of the survey by incorporating practical items addressing participants’ awareness of and expectations regarding specific AI applications, as well as their willingness to allocate a monthly budget to an application perceived as meeting those expectations. In addition, it aimed to broaden the scope from a purely clinical to a more social perspective.

### 4.2. Interpretation of the Findings

The principal finding of this study is that oral and maxillofacial radiology training and experience did not produce a uniform enthusiasm gradient for AI—and that, on reflection, is perhaps the more informative finding. Rather than simply growing more positive at each stage, groups with greater specialty exposure demonstrated lower present-day composite scores alongside a significantly stronger anticipated shift toward near-future acceptance. The observed pattern should be interpreted as a difference in self-reported attitudes and expectations rather than as evidence of directly measured AI literacy. Groups with greater OMFR exposure reported lower present-day acceptance but a larger anticipated shift toward near-future AI use. This may indicate a more cautious evaluation of current AI applications; however, the present study did not directly assess AI literacy, critical appraisal ability, or knowledge of AI governance.

This reading fits reasonably well with what the recent survey literature has reported. Across studies from Türkiye, India, Brazil, South Korea, Saudi Arabia, and elsewhere, dental students and practitioners have generally expressed positive views about AI while also showing meaningful variation tied to educational background and seniority [8,9,10,11,12,13,14,15,16,17,18,19,20,21,22,31,32,33]. A recurrent pattern—confirmed here—is that near-future optimism consistently outpaces present-day enthusiasm [8,9,10,11,12,13,14,15,16,17,18,19,20,21,22,34]. In the paired analyses, all five near-future domains were rated more favorably than their present-day counterparts, with the largest gains appearing for anamnesis/systemic findings and inspection-based evaluation. This pattern mirrors what Bisdas et al. found in their multinational student survey [35].

While participants expressed growing acceptance for AI-driven diagnostic assistance, a clear distinction remains between perception toward tools that augment clinical decision-making and those that operate with full autonomy. Our findings suggest that dental professionals view AI primarily as a collaborative decision-support system, maintaining the clinician’s role as the final arbiter of diagnostic responsibility, rather than a replacement for professional judgment in autonomous decision-making processes [19].

The present data also add texture to the oral-radiology-specific literature. Earlier surveys in this space found high interest in AI among students and dentists engaged with oral radiology, and repeatedly underlined the need for formal training [9,10,11,12,13,32]. What the present findings add is a note of qualified caution: specialty-exposed groups were relatively more reserved about certain present-day applications—particularly inspection-related and laboratory-related tasks. Rather than representing a retreat from optimism, this seems better understood as professional calibration. Specialty-exposed respondents were relatively more reserved about certain present-day applications, particularly inspection-related and laboratory-related tasks. This should not be interpreted as direct evidence that these participants had greater workflow awareness, medicolegal awareness, or critical appraisal ability. Rather, the finding suggests that professional status was associated with different levels of self-reported acceptance across AI application domains. Potential explanations such as clinical responsibility, workflow demands, interpretability, and governance concerns remain interpretive and should be examined directly in future studies [3,4,13,16,17,18,19,33].

It is also worth noting that professional status remained associated with perceived feasibility for automated radiologic diagnosis and treatment planning, automated anamnesis interpretation, and—most markedly—automated archiving and reporting. These are structured, repetitive, and information-dense tasks, and it is plausible that more experienced practitioners identify them as the more realistic near-term targets for AI, rather than full autonomous diagnosis. This fits with what narrative and systematic reviews have argued about the near-term landscape of dental AI: record management, image triage, segmentation, standardized analysis, and decision support are where clinical integration is likely to happen first [7,16,20,21,33].

The present findings align with the Technology Acceptance Model (TAM), which posits that perceived usefulness and perceived ease of use are critical determinants of technology adoption. In the context of OMFR, this adoption is further moderated by professional status. Present data suggests that while undergraduates may view AI through the lens of ‘perceived usefulness’, residents and specialists apply a ‘professional adaptation’ filter, where ‘perceived ease of use’ is tempered by clinical accountability [36].

Another aspect of the findings is that participants expressed more positive attitudes toward near-future AI applications compared to present-day implementations. This pattern can be interpreted within the framework of technological optimism and professional adaptation. While current tools are often approached with caution due to perceived limitations in reliability and usability, professionals simultaneously anticipate rapid advancements that may enhance clinical utility. Such optimism reflects a broader adaptive process in which healthcare providers critically evaluate existing technologies yet remain open to innovation, thereby facilitating a balanced integration of AI into clinical practice.

Radiology residents exhibited the most cautious present-day attitudes, a finding that warrants careful interpretation. Rather than reflecting a lack of innovation, this ‘tempered optimism’ likely stems from an emerging awareness of the ‘black box’ phenomenon inherent in deep learning models. As residents are currently undergoing rigorous training in diagnostic gold standards, they are uniquely positioned to recognize the gap between AI-driven pattern recognition and clinical ground truth. This skepticism is not a barrier to adoption but rather a necessary professional trait for maintaining ‘human-in-the-loop’ oversight, which remains crucial for systems intended for diagnostic assistance rather than full autonomy [36,37].

Cozmecu et al. described the DISC profiles as distinct behavioral styles relevant to AI adoption in dentistry. The Dominance (D) profile is results-oriented, decisive, and risk-taking, often viewing technology as a competitive advantage. The Influence (I) profile emphasizes sociability, enthusiasm, and persuasion, acting as a driver of change and communication. The Steadiness (S) profile values stability, collaboration, and team cohesion, contributing to process consistency and supportive integration. Finally, the Conscientiousness (C) profile is detail-focused, rule-driven, and evidence-based, making it essential for data governance and quality assurance [36]. In the present study, greater training and experience in oral and maxillofacial radiology (OMFR) were associated with a more skeptical stance toward current AI applications, yet simultaneously with a markedly more optimistic outlook regarding potential near-future developments. This pattern suggests that advanced OMFR education and clinical exposure may a balanced perspective, mediating between divergent profiles of attitudes toward artificial intelligence and thereby supporting a more nuanced integration of emerging technologies into practice.

Of all the item-level findings, the professional gradient in Craniocatch awareness may be the most telling. No direct comparative study establishes that Craniocatch is universally better recognized than other dental AI platforms, but the pattern seen here has a coherent explanation. Survey evidence consistently shows that hands-on familiarity with specific AI tools increases with educational and professional seniority rather than simply with general AI enthusiasm [38,39]. Craniocatch, specifically, has appeared in multiple peer-reviewed imaging studies from Türkiye—on automatic tooth detection and numbering, panoramic diagnostic charting, cephalometric landmark detection, and alveolar bone loss assessment [40,41,42,43]. These are not niche applications; they sit at the core of what OMFR residents and specialists encounter in training and practice. Encountering such work in the literature, in departmental seminars, or during clinical rotations produces a different kind of familiarity than the more abstract AI literacy that undergraduates typically develop. The gap between conceptual endorsement and practical tool knowledge has been noted repeatedly in recent surveys, where respondents supported AI in principle while reporting limited direct exposure [8,14,15,16,17]. The present findings reinforce arguments for curricula that move beyond definitional familiarity toward active engagement with specific systems, their validation evidence, and their operational limitations [18,21,22,25,26].

Two supplementary findings are worth flagging for their more practice-oriented implications. Tool awareness was concentrated: Craniocatch and, to a lesser degree, Diagnocat were reasonably recognized, while the remaining platforms—Pearl, Overjet, and V7—were largely unfamiliar. This uneven distribution suggests that general awareness of AI has outpaced knowledge of the actual tools available in the field. The strong professional gradient for Craniocatch recognition reinforces the view that specialty training shapes not just general attitudes but also tool-specific familiarity. It should be clearly acknowledged that the study included only participants from Türkiye and that Craniocatch, one of the evaluated applications, was developed locally. Consequently, the component of the study specifically addressing artificial intelligence applications may carry a more regional significance, and interpretations should be made with this contextual consideration in mind.

Willingness to allocate a budget to AI, by contrast, was associated primarily with sex rather than professional status, suggesting that economic readiness for AI adoption may follow a somewhat different logic from educational and specialty-based awareness.

A few aspects of the present study merit particular emphasis. First, the design follows the OMFR training gradient more granularly than most prior work. Grouping respondents into broad categories such as “students” and “dentists” obscures the attitudinal shifts that accumulate across successive stages of specialty formation, and the present design was deliberately structured to avoid that flattening. Second, and perhaps more importantly, the study reframes what positive AI attitudes should mean in an OMFR context. More experienced respondents were not the most uniformly enthusiastic—in several respects, they were the most measured. That kind of discriminating response profile may actually be more desirable from an educational standpoint than undifferentiated enthusiasm. Third, the findings have direct curricular relevance. There is growing consensus in the educational literature that AI competence in dentistry should be staged and discipline-specific, rather than delivered as a single introductory module [9,20,21,26,43]. By mapping how attitudes and awareness change across the OMFR training continuum, the present results offer an empirical foundation for that kind of graduated approach.

At first glance, one might assume a direct bias suggesting an inverse relationship between age and favorable attitudes toward AI. However, closer examination indicates that the association is more complex. Variables such as the intended purpose of the AI tool, the prerequisite knowledge required, its ease of use, and the extent to which it simplifies daily tasks all contribute to shaping perceptions, thereby diminishing age as a negative factor in this relationship [44]. In their study, Cozmecu et al. examined this issue in two distinct dimensions: chronological age and years of professional practice. They reported a negative correlation between age and willingness to adopt new AI applications, whereas professional experience was positively correlated up to ten years but negatively correlated thereafter [45]. In the present study, age increased in parallel with radiology education and professional experience. As a result, the potentially positive influence of age on attitudes toward AI, combined with the heightened interest in AI applications observed among younger participants, produced a balancing effect, rendering age statistically insignificant in determining overall attitudes toward AI.

Among demographic variables, sex emerged as an independent predictor only of budget willingness and the composite change score. The sex-related difference observed for budget willingness should be interpreted cautiously. Although male respondents more frequently selected the highest budget category, the present study was not designed to determine why this difference occurred. Willingness to pay for AI may be influenced by unmeasured factors such as disposable income, career stage, private-practice exposure, practice ownership, patient volume, institutional versus personal payment expectations, responsibility for software purchasing, and previous experience with subscription-based digital tools. Since these variables were not collected, the finding should not be interpreted as evidence of an intrinsic sex-based difference in AI adoption. Rather, it suggests that economic readiness for AI integration may be shaped by professional and socioeconomic factors that require targeted investigation in future studies.

In this cohort, professional context appeared to matter considerably more than demographic background for shaping AI attitudes. This is not an isolated pattern: the broader perception literature has frequently found educational and professional environment to be more stable determinants of AI attitudes than chronological age [13,14,15,16,17,31,32].

### 4.3. Strengths and Limitations of the Study

This study possesses several notable strengths. The sample size exceeded the a priori power requirement, the design encompassed multiple educational and specialty levels within a unified analytical framework, and the statistical approach integrated paired comparisons, reliability testing, composite-score regression, and secondary item-level modeling.

Nonetheless, several limitations should also be acknowledged. The cross-sectional design precludes causal inference, and the findings reflect self-reported perceptions rather than actual AI use, competence, or diagnostic performance. Although the questionnaire showed good internal consistency, it was designed as a domain-oriented survey rather than a formal psychometric scale; the composite scores should therefore be read with some caution.

The cross-sectional design also inherently constrains the ability to draw causal inferences or track the temporal evolution of AI-related attitudes [46]. While present data tries to provide a comprehensive snapshot of attitudes across different educational stages, it cannot discern whether these shifts represent a longitudinal change within individuals as they progress through their careers or merely reflect generational and cohort-specific differences. Consequently, the observed association between specialty experience and attitude shifts remains correlational; future longitudinal studies are essential to determine the causal impact of specific AI training curricula on the long-term professional adaptation of clinicians.

Other limitation is that the sample was drawn from a single national context, and the professional groups were unequally sized, which may limit generalizability and reduce precision in some subgroup comparisons. The potential effects of unequal number of participants in subgroups were addressed by employing Welch ANOVA together with Games–Howell post hoc tests, rather than relying on one-way ANOVA with Tukey’s post hoc procedure. This adjustment avoided the need to randomly exclude participants, which would reduce the statistical power of the study.

While the reported internal consistency (Cronbach’s alpha > 0.85) provides strong evidence for reliability, we acknowledge that self-reported attitude surveys may be subject to social desirability bias.

The voluntary online format may have introduced selection bias, and relevant factors such as prior AI training, direct software exposure, digital literacy, and institutional access to AI tools were not measured. Finally, awareness of specific platforms may also reflect local visibility and regional dissemination patterns. Future multicenter studies with objective measures of AI literacy and documented software use would address several of these gaps.

The study included participants with varying levels of OMFR education and experience who were thought to be more closely related to AI applications. However, general dentists and physicians from other specialties were excluded to clarify this relationship. A recent study also found a higher association between professional experience and education regarding the capabilities and potential integration of AI modalities into workflows in the near future compared to other peers. However, this comparison was not included as a limitation in the current study [36].

On balance, the present findings suggest that AI education in dentistry is staged and discipline-sensitive. Undergraduate learners may benefit most from foundational literacy and realistic orientation, whereas radiology trainees and specialists may benefit from more advanced training in validation, workflow integration, regulation, and responsible implementation. AI competence in dentistry should encompass not only awareness of potential benefits, but also the ability to judge whether a given tool is trustworthy, clinically relevant, and appropriately governed [13,16,17,18,19]. Evidence on AI integration in OMFR training is still limited, and most studies focus on clinical benefit. Information regarding physicians’ software knowledge and feedback related to AI-related budget allocation has rarely been studied in research [47].

## 5. Conclusions

Professional training and experience in oral and maxillofacial radiology is associated with AI-related attitudes in ways that go beyond a simple enthusiasm-versus-caution divide; the null hypothesis of the study is rejected. Groups with greater specialty exposure were more reserved about certain present-day applications, yet demonstrated a stronger anticipated shift toward near-future acceptance—particularly for structured, automation-oriented tasks. For dental and oral radiology curricula, these findings support a staged and discipline-sensitive approach to AI education, one that fosters realistic expectations, critical appraisal skills, and a working understanding of responsible implementation. Beyond general attitudes, the results suggest that OMFR training and experience are also tied to familiarity with specific AI applications, while financial willingness to adopt AI appears to be shaped by partly different factors. Ultimately, sustainable AI integration in dentistry will require more than attitudinal readiness—it will depend on grounded familiarity with validated tools and on addressing the practical and economic realities of clinical implementation.

## Figures and Tables

**Table 1:** Questions and options of the survey applied. Likert scale scores with five options 0–4.

Item	Short Question Text	Option 1 (0)	Option 2 (1)	Option 3 (2)	Option 4 (3)	Option 5 (4)
Q1	Current view: AI in radiologic examinations	Never use	Rare support	Use half-and-half	Mostly AI, I intervene rarely	Trust AI completely
Q2	Current view: AI in inspection-based examinations	Never use	Rare support	Use half-and-half	Mostly AI, I intervene rarely	Trust AI completely
Q3	Current view: AI in laboratory test evaluation	Never use	Rare support	Use half-and-half	Mostly AI, I intervene rarely	Trust AI completely
Q4	Current view: AI in recording/interpreting history and systemic findings	Never use	Rare support	Use half-and-half	Mostly AI, I intervene rarely	Trust AI completely
Q5	Current view: AI in archiving/statistical analysis of demographic and clinical data	Never use	Rare support	Use half-and-half	Mostly AI, I intervene rarely	Trust AI completely
Q6	Near-future view: AI in radiologic examinations	Never use	Rare support	Use half-and-half	Mostly AI, I intervene rarely	Trust AI completely
Q7	Near-future view: AI in inspection-based examinations	Never use	Rare support	Use half-and-half	Mostly AI, I intervene rarely	Trust AI completely
Q8	Near-future view: AI in laboratory test evaluation	Never use	Rare support	Use half-and-half	Mostly AI, I intervene rarely	Trust AI completely
Q9	Near-future view: AI in recording/interpreting history and systemic findings	Never use	Rare support	Use half-and-half	Mostly AI, I intervene rarely	Trust AI completely
Q10	Near-future view: AI in archiving/statistical analysis of demographic and clinical data	Never use	Rare support	Use half-and-half	Mostly AI, I intervene rarely	Trust AI completely
Q11	Feasibility: AI auto-generates radiologic findings, diagnoses, and treatment plans	Impossible	Unlikely	Undecided	Most likely	Absolutely possible
Q12	Feasibility: AI auto-generates diagnoses/plans from inspection images of soft tissues	Impossible	Unlikely	Undecided	Most likely	Absolutely possible
Q13	Feasibility: AI auto-analyzes laboratory tests and creates treatment plans	Impossible	Unlikely	Undecided	Most likely	Absolutely possible
Q14	Feasibility: AI records/interprets history and systemic findings via speech recognition	Impossible	Unlikely	Undecided	Most likely	Absolutely possible
Q15	Feasibility: AI archives clinical data and generates patient-based statistical reports	Impossible	Unlikely	Undecided	Most likely	Absolutely possible
Q16a	Expected best AI performance: radiologic evaluation	Student level	Dentist level	Specialist level	Faculty level	Higher than professor
**Q16b**	Expected best AI performance: inspection-based examination	Student level	Dentist level	Specialist level	Faculty level	Higher than professor
**Q16c**	Expected best AI performance: laboratory result evaluation	Student level	Dentist level	Specialist level	Faculty level	Higher than professor
**Q16d**	Expected best AI performance: history taking/interpreting systemic findings	Student level	Dentist level	Specialist level	Faculty level	Higher than professor
**Q16e**	Expected best AI performance: archiving/statistical reporting of demographic and clinical data	Student level	Dentist level	Specialist level	Faculty level	Higher than professor
**Q17a–e**	Awareness of Diagnocat (a), Craniocatch (b), Pearl (c), Overjet (d), V7 (e)	Never heard of it	Heard of it, not as AI	Heard of it as AI, do not know exact use	Know it, not used	Used it
**Q18**	Monthly budget for an AI application that fully meets expectations	None	$1–10	$11–50	$51–100	$100+

**Table 2:** Participant characteristics.

Characteristic	Value	Response Rate %
Total sample, *n*	234	85.1 (234/275)
Female, *n* (%)	140 (59.8)	93.3 (140/150)
Male, *n* (%)	94 (40.2)	75.2 (94/125)
Age, mean ± SD	26.89 ± 6.23	
Age, median (range)	24 (21–56)	
Fourth-year students, *n* (%)	63 (26.9)	90 (63/70)
Fifth-year students, *n* (%)	78 (33.3)	97.5 (78/80)
Radiology residents, *n* (%)	47 (20.1)	72.3 (47/65)
Radiology specialists/faculty, *n* (%)	46 (19.7)	76.6 (46/60)

**Table 3:** Power analysis and reliability summary.

Measure	Value
A priori minimum sample size	181
Final analyzed sample	234
Estimated achieved power	0.902
Cronbach’s alpha, present-attitude block (Q1–Q5)	0.852
Cronbach’s alpha, near-future-attitude block (Q6–Q10)	0.872

**Table 4:** Descriptive statistics indicating a progressive trend toward more favorable perceptions of AI integration as clinical experience and educational level increased.

Group	Present AI Attitudes	Near-Future AI Attitudes	Expectations	Awareness of Software	Budget	General Score
4th grade	1.34	1.76	2.64	1.15	0.92	1.56
5th grade	1.82	2.12	2.94	1.31	1.12	1.86
OMFR res.	2.18	2.74	3.22	2.05	1.78	2.39
OMFR spc.	2.58	3.12	3.65	2.38	2.08	2.76

**Table 5:** Welch ANOVA and Games–Howell post hoc tests performed.

Welch ANOVA (Overall)	F Value		*p*-Value *
	9.84		0.001 *
Games–Howell Post Hoc	Mean Difference	Std. Error	*p*-Value *
4th grade vs. 5th grade	−0.30	0.19	0.421
4th grade vs. OMFR res.	−0.83	0.17	<0.001 *
4th grade vs. OMFR spc.	−1.20	0.18	<0.001 *
5th grade vs. OMFR res.	−0.53	0.21	0.068
5th grade vs. OMFR spc.	−0.90	0.22	0.003 *
OMFR res. vs. OMFR spc.	−0.37	0.19	0.245

* *p* < 0.05 presents statistical significance.

**Table 6:** Paired comparison of present-day and near-future AI attitudes using the Wilcoxon signed-rank test. Zero-difference pairs were excluded. The reported W statistic corresponds to the smaller of the positive and negative signed-rank sums; therefore, relatively low W values are expected in the presence of many ties and a strongly directional shift.

Domain	Present Mean	Near-Future Mean	Mean Difference	Wilcoxon W	*p* *
Radiologic examination	1.585	1.962	0.376	836	<0.001 *
Inspection-based examination	1.265	1.714	0.449	767	<0.001 *
Laboratory evaluation	1.748	2.145	0.397	525	<0.001 *
Anamnesis/systemic findings	1.722	2.235	0.513	363	<0.001 *
Archiving/statistics	2.107	2.423	0.316	598.5	<0.001 *

* *p* < 0.05 presents statistical significance.

**Table 7:** Multiple linear regression models for composite attitude scores.

Outcome	Predictor	Statistic	*p* *
Present-attitude composite	Professional status/training	F(3, 228) = 3.45	0.017 *
Present-attitude composite	Sex	F(1, 228) = 0.45	0.503
Present-attitude composite	Age	F(1, 228) = 2.68	0.103
Near-future composite	Professional status/training	F(3, 228) = 1.53	0.208
Near-future composite	Sex	F(1, 228) = 0.88	0.349
Near-future composite	Age	F(1, 228) = 2.07	0.152
Change score (near-future minus present)	Professional status/training	F(3, 228) = 7.37	0.003 *
Change score (near-future minus present)	Sex	F(1, 228) = 4.28	0.040 *
Change score (near-future minus present)	Age	F(1, 228) = 0.05	0.818

* *p* < 0.05 presents statistical significance.

**Table 8:** Item-level findings remaining significant after FDR correction and proportional odds assumption assessment using a parallel-lines/Brant-type test.

Outcome	Factor	FDR q	Paralel Lines—Brant Type *p* **
Present-day inspection-related attitude (Q2)	Professional status/training	0.020 *	0.628
Present-day laboratory-evaluation attitude (Q3)	Professional status/training	0.020 *	0.347
Near-future inspection-related attitude (Q7)	Professional status/training	0.034 *	0.585
Perceived feasibility of automatic radio-diagnosis/treatment planning (Q11)	Professional status/training	0.005 *	0.678
Perceived feasibility of anamnesis recording/interpretation (Q14)	Professional status/training	0.028 *	0.886
Perceived feasibility of automatic archiving/reporting (Q15)	Professional status/training	<0.001 *	0.919
Awareness of Craniocatch	Professional status/training	<0.001 *	0.983
Willingness to allocate a budget to AI	Sex	0.028 *	0.126

* q < 0.05 refers to statistical significance. ** *p* > 0.05 describes acceptable assumption.

**Table 9:** Awareness of selected dental AI applications.

Comparison	Mean Difference	*p*-Value	Interpretation
ANOVA (overall)	–	<0.001 *	Significant differences among AI tools
Craniocatch vs. Diagnocat	0.20	0.18	Not significant
Craniocatch vs. Pearl	1.00	<0.001 *	Craniocatch significantly higher
Craniocatch vs. Overjet	0.88	<0.001 *	Craniocatch significantly higher
Craniocatch vs. V7	1.05	<0.001 *	Craniocatch significantly higher
Diagnocat vs. Pearl	0.80	<0.001 *	Diagnocat significantly higher
Diagnocat vs. Overjet	0.68	<0.001 *	Diagnocat significantly higher
Diagnocat vs. V7	0.85	<0.001 *	Diagnocat significantly higher
Pearl vs. Overjet	−0.12	0.42	Not significant
Pearl vs. V7	0.05	0.61	Not significant
Overjet vs. V7	0.17	0.73	Not significant

* *p* < 0.05 presents statistical significance.

**Table 10:** Distribution of Likert scores and mean scores of professional status/experiment groups about awareness for selected dental AI applications with FDR correction.

AI Tool	Mean	0	1	2	3	4	Stu4	Stu5	OMFR Res.	OMFR Spcl/Fac. Member	*p*	FDR q
Diagnocat	1.15	108	36	40	48	2	0.76	0.79	1.49	1.91	0.075	0.151
Craniocatch	1.35	120	15	16	63	20	0.38	0.45	2.28	3.26	<0.001	<0.001 *
Pearl	0.35	189	16	20	9	0	0.22	0.40	0.47	0.35	0.568	0.615
Overjet	0.47	176	20	26	11	1	0.59	0.50	0.43	0.28	0.866	0.866
V7	0.30	196	13	17	8	0	0.24	0.32	0.38	0.28	0.851	0.866

Note: Awareness was coded from 0 to 4 as follows: 0 = never heard of it; 1 = heard of it but did not know it was an AI application; 2 = heard of it as AI, do not know its exact use; 3 = know it but have not used it; 4 = used it. * q < 0.05 values describe statistical significance.

**Table 11:** Willingness to allocate a budget to an AI application and ordinal model summary.

Budget Category	*n* (%)	Female *n* (%)	Male *n* (%)
Would never allocate	12 (5.1)	5 (3.6)	7 (7.4)
$1–10/month	34 (14.5)	26 (18.6)	8 (8.5)
$11–50/month	81 (34.6)	54 (38.6)	27 (28.7)
$51–100/month	52 (22.2)	38 (27.1)	14 (14.9)
$100+/month	55 (23.5)	17 (12.1)	38 (40.4)
**Predictor**	***p*** **value**	**FDR q**	**Interpretation**
Professional status/training	0.134	0.232	Not significant
Sex	0.001	0.028 *	Male vs. female OR 2.32 (95% CI 1.39–3.86)
Age	0.782	0.924	Not significant

* q < 0.05 describe statistical significance.

## Data Availability

The data presented in this study are available on request from the corresponding author. The data are not publicly available due to privacy.

## Abbreviations

The following abbreviations are used in this manuscript:

AI	Artificial Intelligence
EU	European Union
OMFR	Oral and Maxillofacial Radiology
FDR	False Discovery Rate
Q	Question
TAM	Technology Acceptance Model
